# Uniaxial pressure induced stripe order rotation in La_1.88_Sr_0.12_CuO_4_

**DOI:** 10.1038/s41467-022-29465-4

**Published:** 2022-04-04

**Authors:** Qisi Wang, K. von Arx, D. G. Mazzone, S. Mustafi, M. Horio, J. Küspert, J. Choi, D. Bucher, H. Wo, J. Zhao, W. Zhang, T. C. Asmara, Y. Sassa, M. Månsson, N. B. Christensen, M. Janoschek, T. Kurosawa, N. Momono, M. Oda, M. H. Fischer, T. Schmitt, J. Chang

**Affiliations:** 1 Physik-Institut, Universität Zürich, Winterthurerstrasse 190, CH-8057 Zürich, Switzerland; 2 Department of Physics, Chalmers University of Technology, SE-412 96 Göteborg, Sweden; 3 Laboratory for Neutron Scattering and Imaging, Paul Scherrer Institut, CH-5232 Villigen PSI, Switzerland; 4 State Key Laboratory of Surface Physics and Department of Physics, Fudan University, Shanghai, 200433 China; 5 Photon Science Division, Swiss Light Source, Paul Scherrer Institut, CH-5232 Villigen PSI, Switzerland; 6 Department of Applied Physics, KTH Royal Institute of Technology, SE-106 91 Stockholm, Sweden; 7 Department of Physics, Technical University of Denmark, DK-2800 Kongens Lyngby, Denmark; 8 Laboratory for Neutron and Muon Instrumentation, Paul Scherrer Institut, CH-5232 Villigen PSI, Switzerland; 9 Department of Physics, Hokkaido University - Sapporo, 060-0810 Sapporo, Hokkaido Japan; 10 Department of Applied Sciences, Muroran Institute of Technology, Muroran, 050-8585 Japan

**Keywords:** Electronic properties and materials, Superconducting properties and materials

## Abstract

Static stripe order is detrimental to superconductivity. Yet, it has been proposed that transverse stripe fluctuations may enhance the inter-stripe Josephson coupling and thus promote superconductivity. Direct experimental studies of stripe dynamics, however, remain difficult. From a strong-coupling perspective, transverse stripe fluctuations are realized in the form of dynamic “kinks”—sideways shifting stripe sections. Here, we show how modest uniaxial pressure tuning reorganizes directional kink alignment. Our starting point is La_1.88_Sr_0.12_CuO_4_ where transverse kink ordering results in a rotation of stripe order away from the crystal axis. Application of mild uniaxial pressure changes the ordering pattern and pins the stripe order to the crystal axis. This reordering occurs at a much weaker pressure than that to detwin the stripe domains and suggests a rather weak transverse stripe stiffness. Weak spatial stiffness and transverse quantum fluctuations are likely key prerequisites for stripes to coexist with superconductivity.

## Abstract (web summary)

Transverse stripe order fluctuations may promote superconductivity, but experimental verifications remain difficult. Here, the authors report that a mild uniaxial pressure changes the ordering pattern and pins the stripe order to the crystal axis in La_1.88_Sr_0.12_CuO_4_.

## Introduction

In the cuprates, stripes of doped holes—forming string-like antiferromagnetic domain walls that periodically modulate the charge density—have been both theoretically proposed^1–8^ and experimentally revealed^9^. The interplay between stripes and superconductivity is crucial^10^. Whereas static stripe order appears detrimental^11,12^, fluctuating stripes may be favourable for superconductivity^13–15^. Transverse stripe fluctuations, for example, have been theoretically suggested to promote superconductivity by enhancing the Josephson coupling between stripes^13^. While such meandering motions of stripes are driven by zero-point fluctuations^13^, they are also subject to a finite spatial stiffness, stemming from Coulomb repulsion and the underlying crystal lattice that defines the direction of the stripe order. In systems like the cuprates^9^, nickelates^16^ and manganites^17,18^, charge-stripe order is thus usually pinned either parallel or diagonally to a principal atomic lattice axis.

In a strong-coupling picture, meandering stems from transverse excitations in the form of kinks shifting the stripes by an integer number of atomic units^19,20^. On a macroscopic level, such kinks depin the stripe from the lattice. It has been suggested that the transverse stripe fluctuations have a crucial effect on the competition between charge order and superconductivity, and lead to a rich phase diagram featuring an electronic solid, an isotropic phase, and in between liquid crystal states coexisting with superconductivity^13^. However, direct experimental studies of charge-stripe dynamics remain challenging^21^. As yet, little is known about the lattice pinning potential and the transition from static to fluctuating meandering stripes.

Among hole-doped cuprate compounds, La_1.88_Sr_0.12_CuO_4_ (LSCO) is unique because the orthorhombic lattice distortion is diagonal to the stripes, providing a less compatible lattice 'host'. In LSCO, the charge order is manifested by eight satellite reflections at **Q** = ***τ*** + **q**_**i**_^22,23^. Here, ***τ*** is a fundamental Bragg peak and **q**_**1,2**_ = ± (*δ*_∥_, *δ*_⊥_) with *δ*_∥_ ≈ 1/4. The transverse incommensurability *δ*_⊥_ ≈ 0.011 is far beyond the expectation from orthorhombic twinning^24–26^. The remaining six reflections appear at a mirror (*q*_*y*_ → − *q*_*y*_) and rotation (*q*_*x*_ → *q*_*y*_) symmetric equivalent positions. In the strong-coupling picture, the charge-stripe order is locally commensurate but with the possibility of phase jumping^19,20,27,28^. The modulation *δ*_⊥_ is understood via kink ordering that effectively rotates the stripes away from the principle crystal axes. LSCO is thus a unique example of charge order “unlocked” from the lattice. As such, LSCO can be viewed as an intermediate stepping stone between statically pinned and fluctuating stripes.

Here, we study the transverse pinning properties of the charge-stripe order. For this purpose, we performed a resonant inelastic x-ray scattering (RIXS) experiment employing a uniaxial pressure application. Our setup enables weak in situ compressive strain along a copper-oxygen bond direction. We show how modest strain application gradually pins the stripe order to the crystal axis along the copper-oxygen bond direction, and thus demonstrate that uniaxial pressure allows tuning of kink ordering in LSCO. We find that the lattice pinning potential is weaker than that to detwin the stripe order. This suggests that, at least in LSCO, transverse stripe fluctuations possess an energy scale relevant for the ground state properties. The recent demonstration of uniaxial pressure tuning of electronic instabilities in combination with RIXS^29,30^ opens a new avenue for spectroscopy studies of quantum materials.

## Results

### Uniaxial pressure effect

**Fig. 1:**
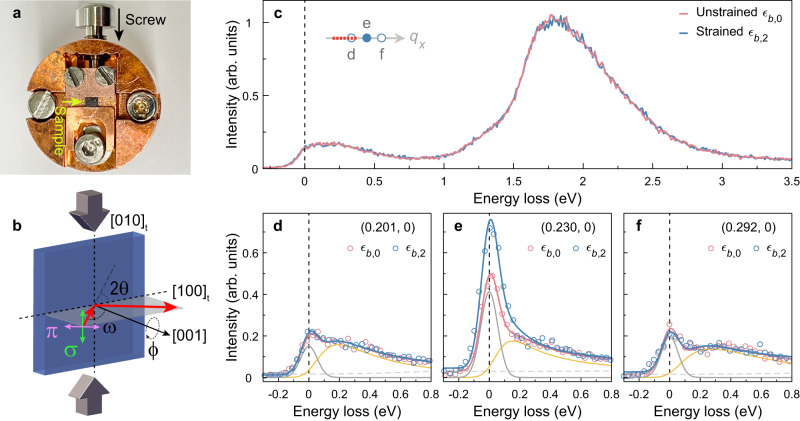
Uniaxial strain tuning of charge-stripe order in La_1.88_Sr_0.12_CuO_4_. **a** Photograph of the strain device—sample (black rectangle) and in situ mechanical screw mechanism are indicated with arrows. **b** Illustration of the scattering geometry with respect to the uniaxial strain application. Vertical (*σ*) and horizontal (*π*) linear light polarisations are indicated with respect to the scattering plane. **c** RIXS spectra including high-energy excitations recorded with (blue, *ϵ*_*b*,2_) and without (red, *ϵ*_*b*,0_) strain for *T* ≈ 28 K. The inset indicates the momentum along **Q** = (*q*_∥_, 0). The red dotted line reveals the momentum integration used for RIXS spectra in **c** and circles indicate the momenta for the RIXS spectra in **d**–**f**. **d**–**f** Low-energy part of RIXS spectra recorded with and without strain for momenta as indicated. Line profiles in **d**–**f** represent fits including a polynomial background (grey dashed line), a damped harmonic oscillator to model the paramagnetic contribution (orange line), and a Gaussian line shape covering the elastic scattering (grey solid line). The intensity is given in arbitrary units.

Fig. 1 shows RIXS spectra recorded along the (*q*_∥_, 0) direction with and without strain application. The spectra reveal an elastic and low-energy (≲1 eV) paramagnetic contribution and strong *dd* excitations at higher energy. The *dd* and the spin excitations show no discernible strain effect. This is in contrast to strain experiments on films. In La_2_CuO_4_ thin films, strain *ϵ* = (*a* − *a*_0_)/*a*_0_ of the order ~1% yields a pronounced change of the *dd* excitations. A modification of the *dd* profile is easily detectable even with a strain of *ϵ* ~ 0.1% (ref. ^31^). Similar results are reported on LaCoO_3_ films^32^. Furthermore, the electronic and magnetic excitations in Sr_2_IrO_4_ thin films are sensitive to strain in the order of ~0.2%^33^. The absence of uniaxial pressure effects in our experiment suggests that the crystal field environment is only marginally modified^34^. This is consistent with our strain calibration using x-ray diffraction (XRD) that yields an upper bound of the *c*-axis lattice expansion *ϵ*_*c*_ = (*c* − *c*_0_)/*c*_0_ ≲ 0.015% (see Methods, Supplementary Note 3 and Supplementary Fig. 2), which is at least an order of magnitude weaker than the maximum strain applied in ref. ^35^. Applying Poisson’s ratio^35,36^, we estimate the upper bound of the in-plane compressive strain − *ϵ*_*b*_ = (*b* − *b*_0_)/*b*_0_ applied to be *ϵ*_*b*_ ≲ 0.04%. Data in our work were obtained under three strain conditions with *ϵ*_*b*,0_ = 0 and 0 < *ϵ*_*b*,1_ < *ϵ*_*b*,2_ < 0.04%.

This strain limit stems from the in situ operational screwdriver that provides finite mechanical force due to its magnetic coupling mechanism. Our strain cell (Fig. 1a) thus generates very modest uniaxial pressure. Away from **Q** = (*δ*_∥_, ±*δ*_⊥_), the strain has virtually no effect on the elastic and spin excitation scattering channels. The spectra obtained with and without uniaxial pressure are indistinguishable (Fig. 1d, f). At **Q** = (*δ*_∥_, 0) by contrast, elastic scattering is significantly increased after the application of pressure (Fig. 1e).

### Stripe order (de)pinning

**Fig. 2:**
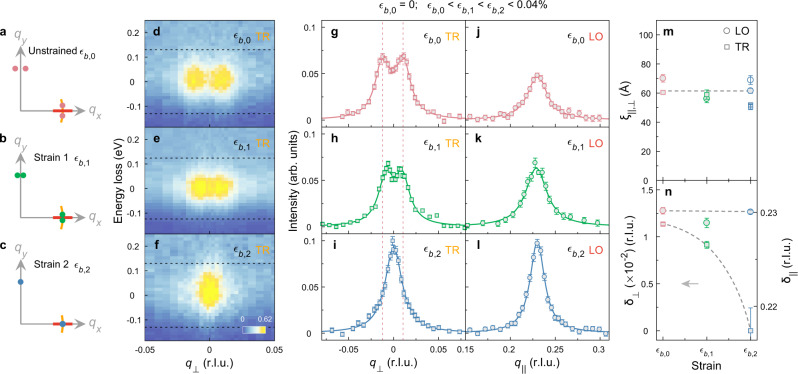
Evolution of charge-stripe order structure in La_1.88_Sr_0.12_CuO_4_ under uniaxial strain. **a**–**c** Illustration of the stripe order diffraction pattern as uniaxial pressure is increased. The splitting of stripe order peaks in **a**, **b** is exaggerated for clarity. Red and orange thick lines denote longitudinal (LO) and rocking (approximately transverse, TR) scans, respectively shown in **g**–**l** for *T* = 28 K ≈*T*_*c*_. **d**–**f** RIXS intensity maps as a function of energy and *q*_⊥_, from which scans in **g**–**i** are obtained. Black dashed lines, in **d**–**f**, mark the energy integration window of elastic intensity. Red dashed lines, in **g**–**i**, indicate the fitted peak positions of the transverse scan at zero strain. **m** Correlation length and **n** incommensurability along longitudinal and transverse directions extracted from fits (solid lines in **g**–**l**) as described in the main text and Supplementary Information. Open symbols in **m** denote results obtained on a repeated measurement with strain value comparable to strain 2. Error bars in **g**–**l** and **m**, **n** are set by counting statistics and standard deviation of fittings, respectively. Uniaxial pressure is increased from strain 1 (*ϵ*_*b*,1_) to strain 2 (*ϵ*_*b*,2_) by mechanically turning the screw pressing onto the sample. Strain 2 corresponds to the strain value for data shown in Fig. 1.

**Fig. 3:**
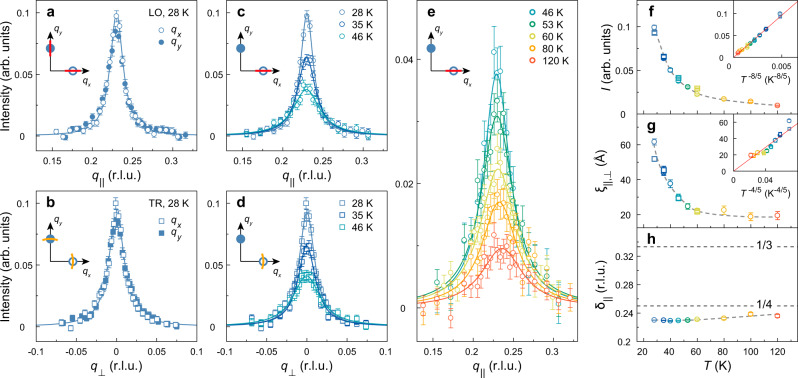
Temperature evolution of lattice pinned charge-stripe order in La_1.88_Sr_0.12_CuO_4_. **a** Longitudinal (circles) and **b** transverse (square) scans through the stripe ordering vectors at *T* ≈ *T*_*c*_. **c**–**e** Temperature dependence of the stripe order diffraction peaks. Solid lines in **a**–**e** are Lorentzian fits from which amplitude, correlation length and incommensurability are inferred and plotted in **f**–**h** versus temperature. Circles and squares in **f**–**h** denote fitting results from the longitudinal and transverse scans, respectively. Error bars in **a**–**e** and **f**–**h** are set by counting statistics and standard deviation of fittings, respectively. Dashed lines in **f**, **g** are guides to the eyes. The two horizontal dashed lines in **h** indicate lattice commensurate values. Red solid lines in the insets of **f**, **g** are linear fits.

RIXS spectra are fitted by modelling elastic and magnon scattering with a Gaussian and a damped harmonic oscillator functional form, respectively^37^ (Fig. 1d–f). The Gaussian width is fixed to the instrumental resolution. Fig. 2d–f displays RIXS intensity as a function of momentum and energy loss. Elastic intensity is obtained by integrating the spectral weight around zero energy within ± FWHM energy window (black dashed lines, see Methods for details). The resulting longitudinal (*q*_∥_, 0) and transverse (*δ*_∥_, *q*_⊥_) scans are plotted in Fig. 2g–l. Under ambient pressure conditions, elastic scattering peaks appear at (*δ*_∥_, ±*δ*_⊥_)^24,38,39^, resulting in a double-peak structure in the transverse scan (Fig. 2d, g). Upon application of uniaxial pressure, *δ*_∥_ remains unchanged (Fig. 2j–l). By contrast, the transverse incommensurability *δ*_⊥_ is highly sensitive to uniaxial pressure and quickly vanishes upon pressure application (Fig. 2g–i). This results in a transverse scan that features a single peak structure centred around (*δ*_∥_, 0). The same effect is found along the perpendicular copper-oxygen bond direction (Fig. 3a, b). Modest uniaxial pressure thus generates a twinned charge order structure with ordering vectors (*δ*_∥_, 0) and (0, *δ*_∥_). As shown in Supplementary Fig. 4, the stripe order remains pinned after releasing uniaxial pressure. It is possible that uniaxial pressure triggers a meta-stable crystal structure which stabilises the pinning of stripe order. Within statistical errors, the correlation lengths (longitudinal *ξ*_∥_ and transverse *ξ*_⊥_) display no change upon application of uniaxial pressure (Fig. 2m). Furthermore, we find isotropic correlation lengths *ξ*_∥_ ≈ *ξ*_⊥_.

### Temperature dependence

After obtaining the pinned charge-stripe order, we studied its temperature evolution with uniaxial strain released. With increasing temperature, the charge order peak amplitude *I* decreases in a *T*^−*η*^ fashion with *η* ≈ 8/5 (Fig. 3c–f) up to the highest measured temperature of 120 K. The correlation length roughly scales with peak amplitude as *I* ~ *ξ*^2^ (see insets of Fig. 3f, g) and saturates around 20 Å in the high-temperature limit. Such a scaling behaviour was also revealed in La_0.165_Eu_0.2_Sr_0.125_CuO_4_^37^ and therefore represents a universal characteristic of charge correlation in stripe-ordered cuprates. Finally, we find that the incommensurability *δ*_∥_ increases only marginally with temperature and never exceeds 1/4 within our probing window.

## Discussion

To discuss the uniaxial pressure-induced stripe (de)pinning effect, we employ both a phenomenological Landau model^40,41^ and a strong-coupling real-space picture. Generally, a two-dimensional charge-density-wave modulation with wave vectors **Q**_*x*_ and **Q**_*y*_ is described by

1 $$\delta \rho ({{{{{{{\bf{r}}}}}}}})={{{{{{{\rm{Re}}}}}}}}({\phi }_{x}{e}^{i{{{{{{{{\bf{Q}}}}}}}}}_{x}\cdot {{{{{{{\bf{r}}}}}}}}})+{{{{{{{\rm{Re}}}}}}}}({\phi }_{y}{e}^{i{{{{{{{{\bf{Q}}}}}}}}}_{y}\cdot {{{{{{{\bf{r}}}}}}}}}),$$

where *δρ*(**r**) is the spatial charge modulation and *ϕ*_*i*_ with *i* = *x*, *y* are amplitudes. The Landau free energy density for these amplitudes in a tetragonal system is given by:

2 $${f}_{0}=	\,{\kappa }_{\parallel }(| {\partial }_{x}{\phi }_{x}{| }^{2}+| {\partial }_{y}{\phi }_{y}{| }^{2})+{\kappa }_{\perp }(| {\partial }_{y}{\phi }_{x}{| }^{2}+| {\partial }_{x}{\phi }_{y}{| }^{2})\\ 	+\alpha (| {\phi }_{x}{| }^{2}+| {\phi }_{y}{| }^{2})+\frac{\beta }{2}{(| {\phi }_{x}{| }^{2}+| {\phi }_{y}{| }^{2})}^{2}-\gamma | {\phi }_{x}{| }^{2}| {\phi }_{y}{| }^{2},$$

where the parameters *α*, *β*, *γ* describe the homogeneous phase, while *κ*_∥_ and *κ*_⊥_ link to the longitudinal and transverse stripe order stiffness. Spontaneous charge order emerges when *α* < 0. Chequerboard and stripe orders are found for *γ* > 0 and *γ* < 0, respectively. The fourfold symmetry implies that both structures are manifested by reflections at **Q**_**1**_ = (*δ*_∥_, 0) and **Q**_**2**_ = (0, *δ*_∥_). An orthorhombic distortion with *B*_1*g*_ or *B*_2*g*_ symmetry adds the following terms to the free energy density^40^:

3 $${f}_{{{{{{{{\rm{orth}}}}}}}}}=-{O}_{ab}{Q}_{a}{Q}_{b}\bar{{\phi }_{a}}{\phi }_{b}+g({O}_{ab}i{Q}_{a}\bar{{\phi }_{b}}{\partial }_{b}{\phi }_{a}+c.c.),$$

where *a*, *b* = *x*, *y* and *O* = *h*_∥_*σ*_3_ or *O* = *h*_⊥_*σ*_1_ represents the *B*_1*g*_ or *B*_2*g*_ symmetry-breaking strain with *h*_∥(⊥)_ and *σ*_*j*_ being the strain magnitude and the Pauli matrices, respectively. *g* is a phenomenological parameter and higher-order terms are neglected.

**Fig. 4:**
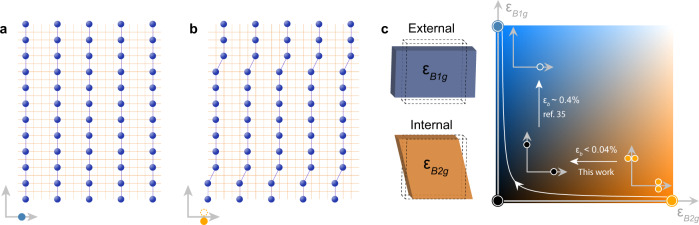
Stripe order structures and strain phase diagram. **a**, **b** Schematic of half-filled, site-centred stripes. The orange grid represents the square CuO_2_ lattice. Spheres illustrate the charge stripes. The in-between antiferromagnetic texture is not shown. **a** illustrates a statically pinned stripe order. Transverse kink ordering is illustrated in **b**. The density of aligned kinks along the stripes determines the transverse incommensurability *δ*_⊥_. For unstrained LSCO, the density is about one kink every 20 copper sites. A schematic of the corresponding diffraction pattern is shown in the bottom-left corner. A dashed open circle indicates the position of charge order peak from the stripe twin domain rotating to the opposite direction. **c** Stripe order phase diagram as a function of orthorhombic *B*_1*g*_ and *B*_2*g*_ strain. The colour code indicates the parameter space coordinates (*gh*_⊥_/*κ*_⊥_, *gh*_∥_/*κ*_∥_), where *gh*_*i*_ with *i* = ∥, ⊥ represent orthorhombic strain and *κ*_*i*_ the stripe stiffness. Blue, black and orange phases represent pinned detwinned, pinned twinned and depinned twinned stripe order, respectively. White curve with arrow indicates schematically the connecting experiment trajectory upon application of uniaxial pressure. The straight white arrows indicate uniaxial pressure (this work and ref. ^35^) applied to connect the different stripe order structures. Schematics on the left-hand side illustrate the two types of orthorhombic lattice distortions.

In absence of external strain, LSCO adopts the low-temperature orthorhombic structure with *B*_2*g*_ symmetry (see Fig. 4c). In this case, *f*_orth_ gives rise to a small rotation of the ordering vector away from the copper-oxygen bond direction with the new ordering vector being $\widetilde{{{{{{{{\bf{Q}}}}}}}}}=| {{{{{{{\bf{Q}}}}}}}}| (1,g{h}_{\perp }/{\kappa }_{\perp })$. Such a transverse incommensuration is indeed observed^24,25^ (Fig. 2d, g). Application of external strain along the copper-oxygen bond direction promotes the *B*_1*g*_ symmetry-breaking terms with magnitude *h*_∥_. For *h*_∥_ ≫ *h*_⊥_, detwinned stripe order pinned to the crystal lattice is expected and recently realised experimentally^35^. With the modest uniaxial pressure applied in this study, we deem that *h*_∥_≤*h*_⊥_. The observation of twinned stripe order with *δ*_⊥_ → 0 suggests that even modest uniaxial pressure induces a space group change of the crystal structure or reduced orthorhombic distortions. A recent numerical calculation using the Hubbard model shows that the stripe rotation is sensitive to the anisotropy of the next-nearest neighbour hopping^42^. This agrees with our finding that modest uniaxial pressure seems to induce an approximate tetragonal crystal field environment. The evolution from twinned depinned to detwinned pinned stripe order is depicted in Fig. 4c.

In the real-space view^19,20,28^, the stripe incommensuration in unstrained LSCO corresponds to a slanted stripe phase with an average angle of ~3^∘^ rotated away from the crystal axis. A microscopic picture of the global incommensuration involves domain walls on stripes as elementary excitations of the order parameter^19^. Since any charge excitation along the stripe results in an increase in Coulomb energy, transverse excitations are therefore energetically favourable if the curvature energy—reflected by the transverse stiffness *κ*_⊥_—is small. The model thus considers one-dimensional stripes as quantum strings with transverse kinks. It has been shown^19^ that inter-string coupling leads to a symmetry-breaking phase with directional kinks and thus slanted stripes. Internal or external *B*_1*g*_ orthorhombic strain could possibly increase the transverse stripe stiffness *κ*_⊥_ through an enhanced electron-phonon coupling^43^. The decrease of the transverse incommensurability *δ*_⊥_ ∝ *gh*_⊥_/*κ*_⊥_ is therefore likely a result of both the reduced *B*_2*g*_ orthorhombicity and enhanced stripe stiffness. On the other hand, the stripe density is reflected by the longitudinal modulation. The observation of longitudinal lattice incommensurability (*δ*_∥_ ≠ 1/4) may suggest the presence of stripe disorder (see Fig. 4a, b and Supplementary Fig. 6). The fact that *δ*_∥_ remains essentially unchanged upon application of modest uniaxial pressure indicates that the disorder potential is weakly dependent on pressure. Application of larger uniaxial pressure is known^35^ to drive *δ*_∥_ → 1/4. In the quantum lattice string model, static stripes melt through a transverse kink proliferation^20,28^, characterised by the transverse fluctuation magnitude^13^. Although the energy and time scales of the stripe dynamics^14,21^ are not directly resolved here, their prominent role is signified by the weak transverse stiffness revealed by our results. The fact that modest external strain stabilises the stripe phase suggests that LSCO is in the vicinity of a quantum melting point. The associated quantum fluctuations are likely crucial to the coexistence of superconductivity and stripe phase.

## Methods

### Samples

La_1.88_Sr_0.12_CuO_4_ single crystals were grown by the floating zone method^11^. The superconducting transition temperature is *T*_*c*_ = 27 K.

### Resonant inelastic x-ray scattering

RIXS experiments were carried out at the ADRESS beamline of the Swiss Light Source (SLS) at the Paul Scherrer Institut^44,45^. The energy resolution—ranging from 124 to 130 meV—is estimated by the full-width-at-half-maximum (FWHM) of the elastic scattering peak from amorphous carbon tape. To enhance charge scattering, most data were taken using linear vertical (*σ*) incident light polarisation with grazing exit scattering geometry. Comparative measurements using horizontal (*π*) incident light polarisation have been performed under identical configurations (Supplementary Fig. 3). Wave vector **Q** = (*q*_*x*_, *q*_*y*_, *q*_*z*_) is labelled in reciprocal lattice units (r.l.u.) of (2*π*/*a*, 2*π*/*b*, 2*π*/*c*), where *a* = *b* = 3.78 Å and *c* = 13.2 Å are the lattice parameters of the high-temperature tetragonal unit cell. *q*_∥_ and *q*_⊥_ denote the longitudinal and transverse components of the in-plane momentum (*q*_*x*_, *q*_*y*_) in r.l.u., respectively. Samples were aligned with *a* and *c* axes in the horizontal scattering plane and *b* axis along the vertical direction. The scattering angle was fixed at 2*θ* = 130^∘^ (see Fig. 1b). In-plane momentum is set by varying the *ω* and *ϕ* angles (see Fig. 1b).

### Uniaxial strain application

To apply uniaxial strain, we adapted a tool previously used to cleave crystals for angle-resolved photoemission spectroscopy experiments^46^. For the RIXS measurements, our LSCO crystals were cleaved using a top-post. Uniaxial pressure was applied at low temperature (~28 K) along a copper-oxygen bond direction through an in situ operational screw mechanism (see Fig. 1a).

### Analysis of RIXS data

RIXS intensities are normalised to the weight of *dd* excitations^37^. Elastic intensity is extracted by integrating the spectral weight around zero energy loss within ± FWHM energy window. We have also analysed the data by defining the area of the fitted Gaussian elastic line as the elastic intensity. The two analysis methodologies yield consistent conclusions (Supplementary Figs. 7, 8). Correlation lengths are defined as the inverse half-width-at-half-maximum.

### X-ray diffraction

XRD measurements were performed at 300 K where the uniaxial strain was applied. Since the elastic constants of the sample^47^ and materials used for the strain cell (type 316 stainless steel^48^ and copper^49^) change only slightly below 300 K, the maximum strain values applied in the RIXS and XRD experiments are comparable.

## Supplementary information

Supplementary Information

## Data Availability

All data that support the findings of this study are available from the corresponding authors upon reasonable request. Source data are provided with this paper.

## Source data

Source Data
