# Supplementary material for: Uniaxial pressure induced stripe order rotation in La1.88Sr0.12CuO4
Source: Nat Commun. 2022 Apr 4;13:1795. doi: 10.1038/s41467-022-29465-4 (PMC8979978; doi:10.1038/s41467-022-29465-4)
Supplement: Supplementary file 1 — Supplementary Information [file 41467_2022_29465_MOESM1_ESM.pdf]

Supplementary Information for
Uniaxial Pressure Induced Stripe Order Rotation in $La_{1.88}Sr_{0.12}CuO_4$

Q. Wang *et al.*

Supplementary Note 1. DETERMINATION OF LONGITUDINAL INCOMMENSURABILITY

To precisely determine the longitudinal incommensurability, we first aligned the crystal by finding the specular condition. The slight offset in specular angle is adjusted by measuring the equivalent charge order reflections at $(\pm\delta_{\|}, 0)$, as shown in Supplementary Fig. 1. In this way, we can determine the longitudinal incommensurability within the error of 0.001 r.l.u..

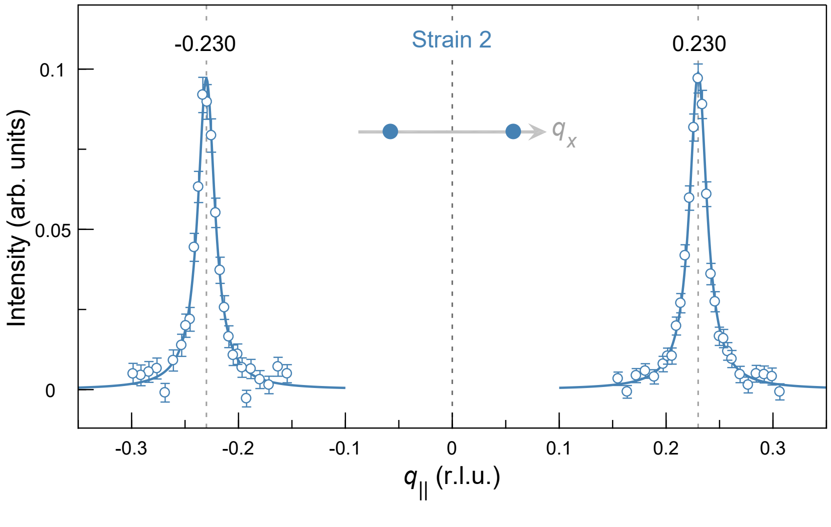

Supplementary Figure 1. Charge order reflections used to determine specular offset. Longitudinal scans through equivalent charge order reflections at $(\pm\delta_{\|}, 0)$ under strain 2.

Supplementary Note 2. FITTING OF CHARGE ORDER DIFFRACTION PEAKS

Under uniaxial strain 2, both longitudinal and transverse scans through the charge order peaks are well described by a Lorentzian profile. At lower strain, the transversely split charge order peaks are described using two two-dimensional Lorentzian functions

$$I = \frac{A_1}{1 + 4(q_{\|} - \delta_{\|})^2/{w_{\|}}^2 + 4(q_{\perp} - \delta_{\perp})^2/{w_{\perp}}^2} + \frac{A_2}{1 + 4(q_{\|} - \delta_{\|})^2/{w_{\|}}^2 + 4(q_{\perp} + \delta_{\perp})^2/{w_{\perp}}^2}, \tag{1}$$

with $w_{\|}$ and $w_{\perp}$ being the width along the longitudinal and transverse directions of each peak and $2\delta_{\perp}$ being the splitting. Transverse scans through the peak positions ($(\pm\delta_{\|}, q_{\perp})$ and $(q_{\perp}, \pm\delta_{\|})$) and longitudinal scans through the valley of the two split peaks ($(q_{\|}, 0)$ and $(0, q_{\|})$) are described respectively by

$$I_{\perp} = \frac{A_1}{1 + 4(q_{\perp} - \delta_{\perp})^2/{w_{\perp}}^2} + \frac{A_2}{1 + 4(q_{\perp} + \delta_{\perp})^2/{w_{\perp}}^2}, \tag{2}$$

and

$$I_{\|} = \frac{2A}{1 + 4(q_{\|} - \delta_{\|})^2/{w_{\|}}^2 + 4\delta_{\perp}^2/{w_{\perp}}^2}, \tag{3}$$

with $A \approx (A_1 + A_2)/2$. By fitting momentum scans along these two directions using the above functions, we obtain the incommensurability $\delta_{\|}$, $\delta_{\perp}$ and correlation length $\xi_{\|}$, $\xi_{\perp}$ as presented in Fig. 2.

Supplementary Note 3. X-RAY DIFFRACTION MEASUREMENT TO DETERMINE THE STRAIN DEPENDENCE OF THE LATTICE PARAMETERS

The nuclear Bragg peaks are not accessible in the RIXS experiment due to kinematic constraints [1]. To calibrate the applied strain, we carried out x-ray diffraction measurements using a Cu Kα x-ray source that allows the access of the $(0, 0, l)$ Bragg peaks with $l = 2, 4, 6, 8$ and 10. X-ray diffraction measurements were performed at 300 K where uniaxial strain was applied. As shown in Supplementary Fig. 2(a-e), θ-2θ scans without and with strain application are fitted with two Lorentzian profiles that account for the diffraction peaks with Cu Kα1 and Kα2 x-rays and a linear background. To extract the c-axis lattice parameter, Bragg peak positions (obtained with Cu Kα1 x-rays) are fitted to the functional form $2\theta = 2\sin^{-1}(\lambda_1 l/(2c)) + 2\theta_0$ (dashed lines in Supplementary Fig. 2f). The fitted c-axis lattice parameter is $c_0 = 13.250(2)$ Å and $c_2 = 13.250(1)$ Å, under unstrained and strain 2 (maximum strain applied in the RIXS experiments) conditions, respectively. The c-axis lattice change is therefore below the measurement precision, based on which we estimate the upper bound of the c-axis lattice expansion $\epsilon_c = (c - c_0)/c_0$ due to the in-plane compression to be $\epsilon_c \lesssim 0.015\%$, which is an order of magnitude smaller than the strain applied in ref. [2].

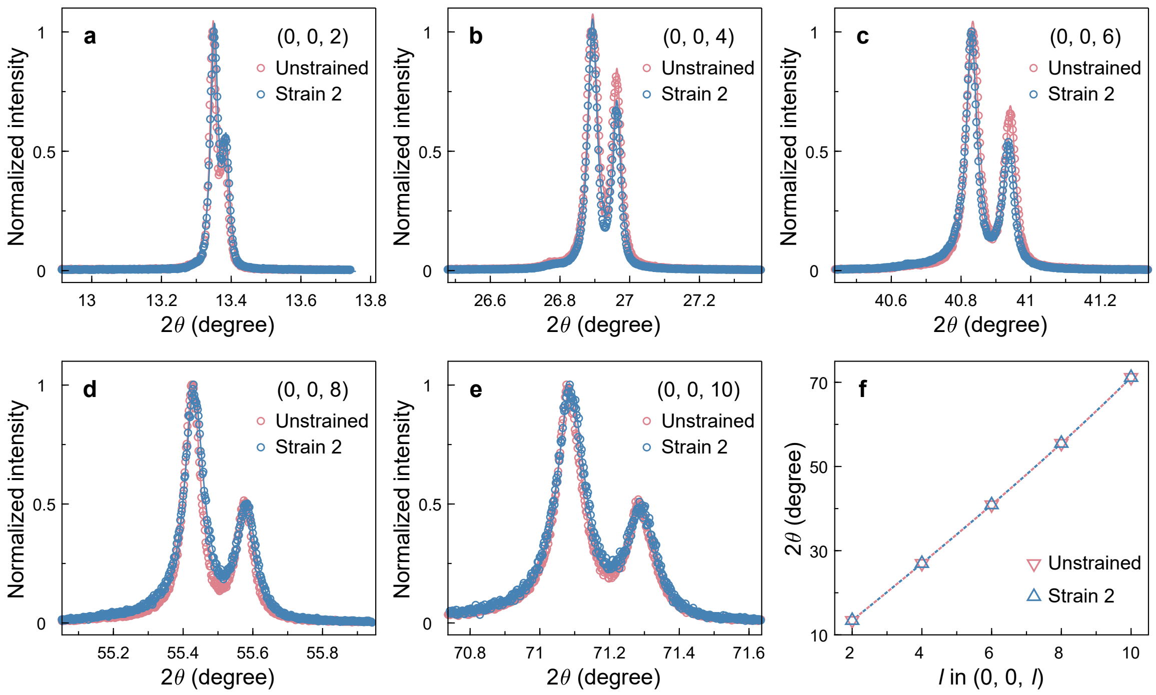

Supplementary Figure 2. Strain dependence of the $(0, 0, l)$ Bragg reflections. (**a-e**) θ-2θ scan of the $(0, 0, l)$ Bragg reflections with $l = 2, 4, 6, 8$ and 10 under zero (red open dots) and strain 2 (blue open dots) measured on a LSCO sample with the same dimensions as those used for the RIXS experiments. The two split peaks originate from Cu Kα1 and Kα2 x-rays with wavelengths $\lambda_1 = 1.5406$ Å and $\lambda_2 = 1.5444$ Å, respectively. Solid lines are fits to two Lorentzian profiles on a linear background. Data are normalized to the maximum intensity. (**f**) Bragg peak positions (obtained with Cu Kα1 x-rays) extracted from (**a-e**). Red and blue dashed lines are least-square fits to the unstrained and strain 2 data as described in the text, respectively.

Supplementary Note 4. POLARISATION DEPENDENCE OF THE CHARGE ORDER INTENSITY

To confirm that the diffraction peaks observed here originate from charge correlations, we measured the charge order diffraction peak before and after strain application with both σ and π incident light polarisations. As shown in Supplementary Fig. 3, consistent momentum structure is revealed by measurements with both polarisations. The intensity ratio between data obtained with two polarisations is consistent with the results on charge order discussed in previous studies [3, 4].

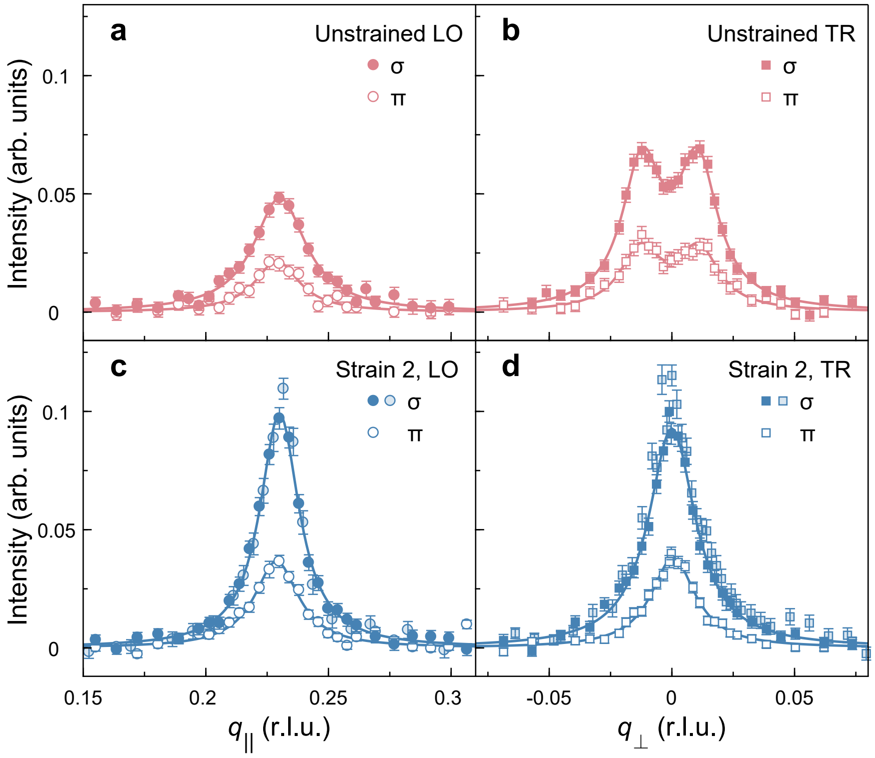

Supplementary Figure 3. **Charge order reflections measured with different incident light polarisations.** Charge order diffraction intensity measured with both σ and π polarised incident x-rays, before (**a,b**) and after (**c,d**) strain application along the longitudinal (**a,c**) and transverse (**b,d**) directions, respectively. Semitransparent symbols represent data obtained in the same experiment as strain 1 data.

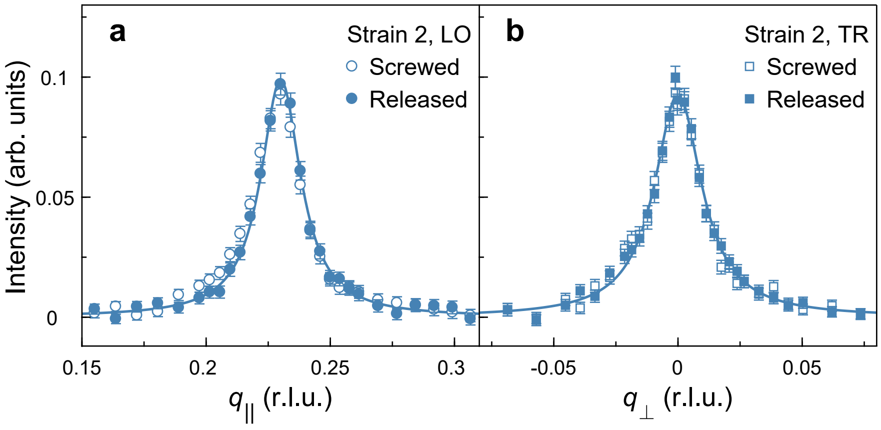

Supplementary Figure 4. Charge order reflections measured before and after screw release at low temperature. Charge order diffraction intensity before (open symbols) and after (filled symbols) screw release measured along the longitudinal (**a**) and transverse (**b**) directions, respectively.

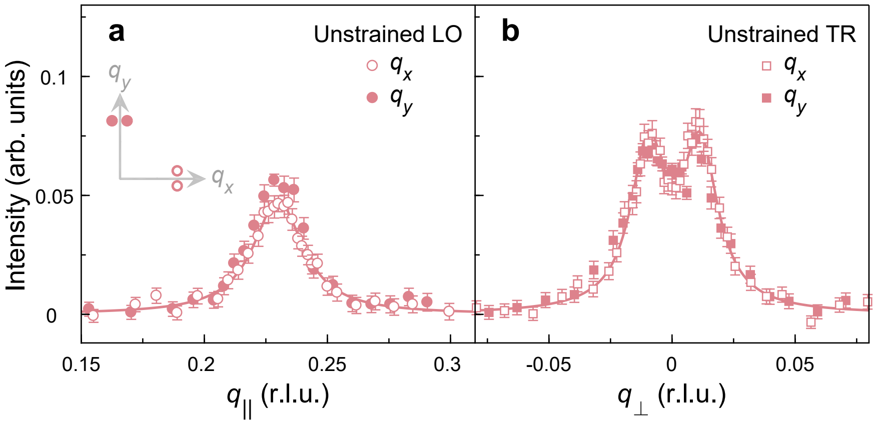

Supplementary Figure 5. Twinning of charge-stripe order without strain. Charge order reflections along q_x (open symbols) and q_y (filled symbols) show comparable profiles. Data were obtained on a different cleave from those presented in the main text. Both the intensity and transverse incommensurability are in good agreement among different samples.

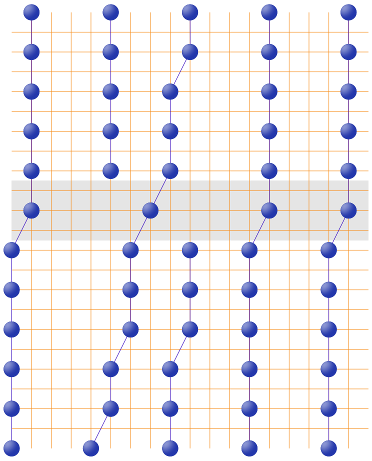

Supplementary Figure 6. Schematic of rotated charge stripes with disorder. Orange grid represents the square CuO_2 lattice. Spheres illustrate the charge stripes. Shaded area marks the stripe disorder that corresponds to a reduced stripe density.

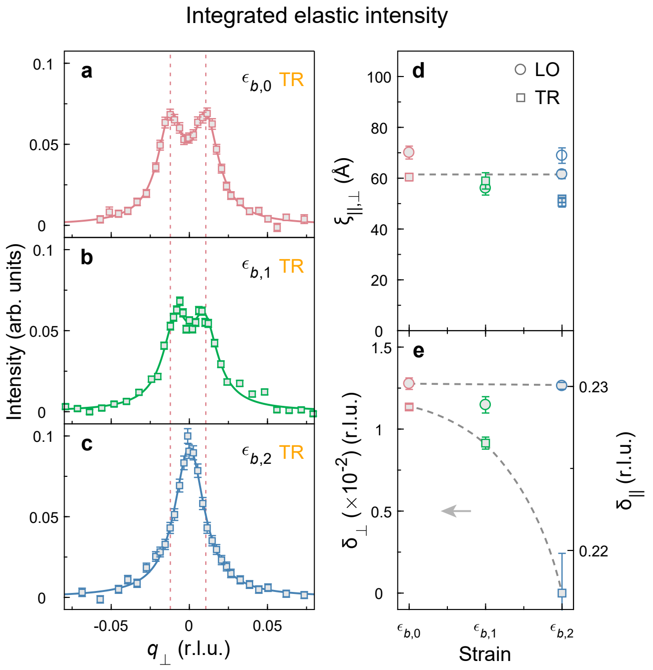

Supplementary Figure 7. **Momentum dependence of charge order obtained with two analysis approaches.** (**a-e**) Transverse **Q**-scans (**a-c**) and their fitting results (**d,e**) with elastic intensity defined by the integration of RIXS intensity over the energy window of ±FWHM, which are the same results as in Fig. 2. (**f-j**) Transverse **Q**-scans (**f-h**) and their fitting results (**i,j**) with elastic intensity defined by the area of fitted elastic peak.

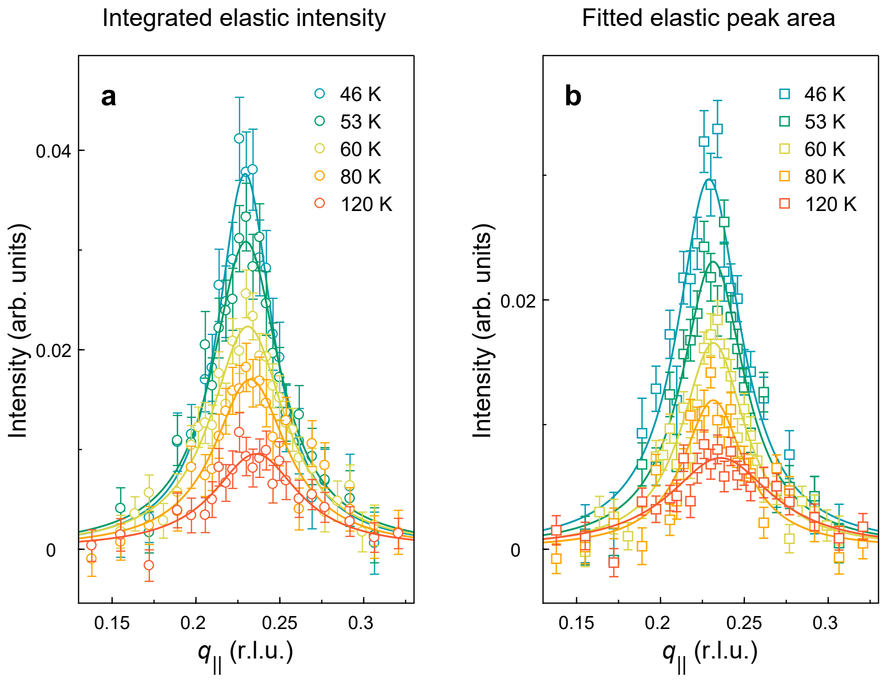

Supplementary Figure 8. **Temperature dependence of charge order obtained with two analysis approaches.** (**a**) Temperature dependence of charge order peak with intensity defined by the integration of RIXS intensity over the energy window of ±FWHM, and (**b**) by the area of fitted elastic peak.

References

1. Boyle, T. J. *et al.* Large response of charge stripes to uniaxial stress in $La_{1.475}Nd_{0.4}Sr_{0.125}CuO_4$. *Phys. Rev. Research* **3,** L022004 (2021).
2. Choi, J. *et al.* Disentangling intertwined quantum states in a prototypical cuprate superconductor. *arXiv:2009.06967.*
3. Wang, Q. *et al.* High-temperature charge-stripe correlations in $La_{1.675}Eu_{0.2}Sr_{0.125}CuO_4$. *Phys. Rev. Lett.* **124,** 187002 (2020).
4. Ghiringhelli, G. *et al.* Long-range incommensurate charge fluctuations in $(Y, Nd)Ba_2Cu_3O_{6+x}$. *Science* **337,** 821–825 (2012).
